# Mechanistic Study of Coffee Effects on Gut Microbiota and Motility in Rats

**DOI:** 10.3390/nu14224877

**Published:** 2022-11-18

**Authors:** Shrilakshmi Hegde, Daniel W. Shi, John C. Johnson, Ramasatyaveni Geesala, Ke Zhang, You-Min Lin, Xuan-Zheng Shi

**Affiliations:** 1 Department of Internal Medicine, University of Texas Medical Branch, Galveston, TX 77555, USA; 2 College of Science, Texas A&M University, College Station, TX 77843, USA; 3 John Sealy School of Medicine Class 2025, University of Texas Medical Branch, Galveston, TX 77555, USA; 4 Department of Pathology, University of Texas Medical Branch, Galveston, TX 77555, USA

**Keywords:** coffee, small intestine, colon, microbiota, motility, smooth muscle

## Abstract

Consumption of coffee has benefits in postoperative ileus. We tested the hypothesis that the benefits may be related to the effects of coffee on gut microbiota and motility and studied the mechanisms of action in rats. The in vitro and in vivo effects of regular and decaffeinated (decaf) coffee on gut microbiota of the ileum and colon were determined by bacterial culture and quantitative RT-PCR. Ileal and colonic smooth muscle contractility was determined in a muscle bath. In the in vivo studies, coffee solution (1 g/kg) was administered by oral gavage daily for 3 days. Compared to regular LB agar, the growth of microbiota in the colon and ileal contents was significantly suppressed in LB agar containing coffee or decaf (1.5% or 3%). Treatment with coffee or decaf in vivo for 3 days suppressed gut microbiota but did not significantly affect gut motility or smooth muscle contractility. However, coffee or decaf dose-dependently caused ileal and colonic muscle contractions in vitro. A mechanistic study found that compound(s) other than caffeine contracted gut smooth muscle in a muscarinic receptor-dependent manner. In conclusion, coffee stimulates gut smooth muscle contractions via a muscarinic receptor-dependent mechanism and inhibits microbiota in a caffeine-independent manner.

## 1. Introduction

Coffee is one of the most popular beverages in the world. Current evidence suggests that it has health benefits in conditions such as cardiovascular diseases (i.e., stroke) [1], Parkinson’s disease [2], metabolic disorders (i.e., Type 2 diabetes) [3], and liver diseases (i.e., non-alcoholic fatty liver disease) [4]. However, the effects of coffee on the gastrointestinal tract (GI) have not been well investigated. Nevertheless, population-based studies found that consumption of coffee is inversely associated with the prevalence of constipation [5,6]. While postoperative ileus is a common condition after abdominal surgeries, recent clinical trials found that postoperative coffee consumption, regardless of caffeine content, reduces the time to first defecation, and reduces the incidence of post-operative ileus and the length of stay in hospital [7,8,9,10]. Some studies suggest that coffee may also exert a protective effect concerning colon cancer [11]. These clinical observations and population-based studies have demonstrated potential benefits of coffee on digestive health. However, what accounts for the benefits of coffee in the GI tract is poorly understood.

The GI tract represents one of the largest interfaces (250–400 m^2^) between the host and environment and is the home of 10^14^ microorganisms, collectively microbiota [12]. Gut microbiota are proposed to play a critical role in host health. On one hand, many commensal bacteria such as *Firmicutes* and *Lactobacillus* are considered as probiotics as their presence in the gut are beneficial to the host [13,14]. On the other hand, some of the microbiota such as *Gamma proteobacteria* and *Enterobacteriaceae* may impose a threat on gut integrity or even play a pathogenic role in gut inflammation and infections [15,16]. When the host consumes coffee, the gut microbiota is among the first to be exposed to it. Unfortunately, the effect of coffee on the gut microbiota has not been well characterized. Jaquet et al. reported [17] that coffee promoted the growth of certain select bacteria species. However, Nakayama et al. [18] found that coffee may exert antibiotic effects, especially towards *E. coli*. There are also reports that fecal bacteria were not affected by coffee consumption [19]. Overall, there is a lack of data concerning the effect of coffee on microbiota as a whole and different groups of potential pathogenic and beneficial bacteria in the gut. As gut microbiota in different parts of the GI tract may have very different functions, the in vivo effect of coffee on the small intestine and colon has not been studied comparatively. It is therefore important to study the effect of coffee on the gut microbiota and determine the in vitro as well as in vivo effects of coffee on total microbiota and different groups of microbes in the small intestine and colon.

Gut motility is crucial to digestive health and homeostasis in the GI tract. Although the autonomic nervous system (mainly the parasympathetic nervous system) is involved in the extrinsic control of gut motility, it is the intrinsic enteric nervous system (ENS) that directly controls gut smooth muscle cells to fulfill the motility function in the gut [20,21]. Earlier manometry studies found that colonic motility was significantly increased in the first 30 min after consuming coffee [22,23]. These in vivo studies focused on the effect of coffee on colon motility. However, the mechanism of action of coffee on gut motility, and whether coffee also has pro-motility properties in the small intestine are not known. These questions are important, as recent clinical observations found that drinking coffee is beneficial for post-operative ileus [7,10], which is a condition largely affecting the small intestine. A better understanding of the mechanism of action of coffee on gut motility may help develop coffee or its components as therapeutics [24,25] for motility disorders such as ileus and constipation. 

We hypothesized that the benefits of coffee in conditions such as constipation, postoperative ileus, and even colorectal cancer may be related to its effects on the gut microbiota and gut motility, two important contributors to maintaining gut homeostasis. We thus undertook a laboratory investigation into the in vitro and in vivo effects of coffee on the gut microbiota and smooth muscle contractility in rats. We also aimed to understand the mechanisms of the effects. Part of the results were presented earlier in an abstract form [26]. 

## 2. Materials and Methods

### 2.1. Preparation of Coffee Solution

The coffee solution was prepared by dissolving 100% *Arabica coffee* powder (Starbucks Corporation, Seattle, WA, USA) in hot water, as described previously [18]. Briefly, boiling water was added to regular or decaffeinated coffee powder (14 mL water for 1 g of coffee powder). The coffee extract solution was mixed well and incubated at room temperature for 15 min. After incubation, the coffee extract solution was centrifuged at 3000 g for 10 min. The supernatant was taken and filtered through a 0.45-µm filter. The filtered coffee solution was used freshly or kept at 4 °C for no more than 3 days. Decaffeinated coffee solution was also prepared in the same way but using 100% Arabica decaffeinated coffee powder (Starbucks Corporation, Seattle, WA, USA). 

### 2.2. Preparations of Intraluminal Fecal Contents and In Vitro Bacteria Growth Assay 

Intraluminal fecal pellets were obtained aseptically from naïve Sprague-Dawley rats (male and female) of 230~280 g. In brief, rats were euthanized with CO_2_, and a laparotomy was operated. The distal ileum (5 cm from the ileal-colon junction) and distal colon (3 cm from the anus) were isolated and opened. Fecal contents were obtained under aseptic operation and transferred to pre-weighed sterile tubes [27]. The sample tubes were kept on ice throughout the procedure. The fecal contents were weighed and mixed well with sterile 1 mL PBS solution (Sigma-Aldrich, St. Louis, MO) and homogenized using a microtube homogenizer (Bel-Art, Wayne, NJ, USA). After homogenization, samples were incubated on ice for 30 min and spun down at 1200 rpm for 3 min. The supernatant collected was used for in vitro bacterial growth inhibition assays.

In the bacterial growth inhibition assay [18], serial dilutions of the supernatant from intraluminal fecal contents isolated from the ileum and colon were plated on regular LB agar and LB agar containing 1.5% or 3.0% (mg/100 mL) coffee. The agar was kept at 37 °C for 24 h. After the incubation, the bacterial colonies on the regular LB agar and coffee-contained LB agar were counted by two independent investigators, and colony forming units (CFUs)/gram sample were calculated.

### 2.3. Oral Gavage Treatment of Rats with Coffee Solution and Fecal and Tissue Preparations

Sprague-Dawley rats (male and female) of 230~280 g (Harlan Sprague Dawley, Indianapolis, IN, USA) were used for the study. The rats were housed in a single cage per rat in a controlled environment (22 °C, 12-h light-dark cycle) and always allowed food and water ad libitum unless stated otherwise. The Institutional Animal Care and Use Committee at the University of Texas Medical Branch approved all procedures performed on the animals. All of the experimental methods were performed in accordance with the Guide for the Care and Use of Laboratory Animals of the National Institutes of Health, USA. 

Rats were randomly assigned into 3 groups, i.e., the vehicle control (Ctr), regular coffee (Coff), and decaffeinated coffee (Decaf) groups. Rats were administered (by oral gavage) [26,28] with distilled water (2 mL), regular coffee (1 g/kg in 2 mL of water), and decaffeinated coffee solution (1 g/kg in 2 mL of water), respectively, daily for 3 consecutive days. At the end of the experimental period, the rats were euthanized. The ileum and colon were opened. The fecal contents of the ileum and colon were collected for microbiology and qPCR studies [27,29,30]. The ileal and colon tissues were taken for measurements of gut smooth muscle contractility [21]. 

### 2.4. Total (Anaerobic and Aerobic) Bacterial Culture from the Colon and Ileum Contents

The total bacterial abundance was quantified by culturing both anaerobic and aerobic bacteria as described previously [26,27,31]. Gifu anaerobic media (GAM) (HIMEDIA, West Chester, PA, USA) and tryptic soy agar (TSA) plates (Difco, BD, Franklin Lakes, NJ, USA) were prepared as described by the manufacturer and stored at 4 °C until use. Fecal samples were collected from the ilium and colon aseptically and were weighed and homogenized immediately in GAM broth by vortexing and using a micro tube homoginizer (Bel-Art, Wayne, NJ, USA). After homogenization, the contents were incubated on ice for 30 min and centrifuged at 1200 rpm for 3 min. Serial dilutions of the supernatant collected were plated on GAM agar plates for enumeration of anaerobic bacteria. Serial dilutions were also plated on TSA plates to quantify aerobic and facultative bacteria. GAM plates were incubated in anaerobic jars containing anaerogen bags (Sigma, St. Louis, MO, USA), whereas TSA plates were incubated in 5% CO_2_ at 37 °C for 24 h. The next day, CFU/gram samples taken were calculated by counting visible bacterial colonies. 

### 2.5. Genomic DNA Extraction and Quantitative RT-PCR Study of Gut Microbiota Abundance

Total genomic DNA was isolated from pre-weighed colon and ilium contents using a QIAGEN stool mini kit according to the manufacturer’s instruction for bacterial DNA isolation. Total bacterial abundance in colon and ilium contents was calculated by absolute quantification of 16S rRNA copies using RT-PCR as described previously [26,27,29,30]. Briefly, 16S rRNA regions were amplified by taking 0.4 µL feces DNA as template, 25 pmol/μL specific primers [UniF: GTGCTGCATGGTCGTCGTCA; UniR: ACGTCGTCCACACCTTCCTC [32] and 1X power SYBR green master mix (Applied Biosystems, Foster City, CA, USA) in 20 μL final reaction volume. All of the PCRs were done in duplicates in a StepOne plus Real-time PCR system (Applied Biosystems, Foster City, CA, USA) [27,29,30]. The PCR conditions were as below: 95 °C for 10 min, followed by 40 cycles at 95 °C for 30 s, 60 °C for 30 s and 72 °C for 45 s. A melting curve analysis was carried out at the end. The standard curve for 16S rRNA quantification was generated by the serial dilution of pJet plasmid (Invitrogen, Waltham, MA, USA) containing 16S rRNA target sequence from *E. coli* (DH10B) [27]. The total bacterial 16S rRNA gene copy numbers/mg of sample was calculated using the following equation: total copy numbers/mg = [mean copy numbers from standard curve X volume of DNA taken (μL)]/[total volume of extracted DNA (μL) X mg of sample used for DNA isolation).

For relative quantification of different classes of bacteria in the ilium and colon contents, below class specific primers were used. *Enterobacteriaceae*: Eco1457F–CATTGACGTTACCCGCAGAAGAAGC, Eco1652R–CTCTACGAGACTCAAGCTTGC (Tm = 61 °C) [33]; *Gamma proteobacteria*: Gamma395f–CCATGCCGCGTGTGTGAA, Gamma871r-ACTCCCCAGGCGGTCTACTTA (Tm = 56 °C) [34]; *Lactobacillus*: F_alllact_IS–TGGATGCCTTGGCACTAGGA, R_alllact_IS–AAATCTCCGGATCAAAGCTTACTTAT (Tm = 58 °C) [35]; *Firmicutes*: S-P-Firm-0352–CAGCAGTAGGGAATCTTC, S-P-Firm-0525–ACCTACGTATTACCGCGG (Tm = 57 °C) [36]. 16S rRNA copies were quantified using UniF and UniR primers as above and used as a reference gene for relative quantification. The reaction consisted of 0.4 µL feces DNA as template, 25 pmol/μL specific primers and 1X power SYBR green master mix (Applied Biosystems, Foster City, CA, USA) in 20 μL reaction. The PCR conditions were 95 °C for 10 min, followed by 40 cycles of 95 °C for 30 s, annealing for 30 s at respective Tm and 72 °C for 45 s. A melting curve analysis was carried out at the end. All of the PCR reactions were done in duplicates in a StepOne plus Real-time PCR system (Applied Biosystems, Foster City, CA, USA) using a temperature gradient block that enabled the use of different annealing temperatures for every primer set. Results are expressed as fold-change in the abundance of different classes of bacteria compared to the total bacterial abundance (16S rRNA copies) and were calculated using the ΔΔCT method. 

### 2.6. In Vivo Gut Motility Study

Colonic propulsive motility was measured as described previously [37,38]. Rats treated with saline, regular coffee and decaffeinated coffee for three days were lightly anaesthetized by isoflurane. A 6 mm-diameter glass bead was inserted into the distal colon about 3 cm from the anus of each rat. After bead insertion, the rats were replaced individually without food and water in their home cages. The time required for expulsion of the glass bead, the mean expulsion time, was determined for each rat. 

### 2.7. Intestinal and Colonic Muscle Contractility Study

Tissue samples of 2 cm-long distal ileum (5 cm from the ileal-colon junction) and colon (2 cm from the anus) were collected and placed immediately in carbogenated Krebs buffer (in mmol/L: 118 NaCl, 4.7 KCl, 2.5 CaCl_2_, 1 NaH_2_PO_4_, 1.2 MgCl_2_, 11 D-glucose, and 25 NaHCO_3_) [39,40,41]. The ileal and colonic specimens were opened along the mesenteric border and pinned flat in a Petri dish with Sylgard base in a carbogenated Krebs buffer. Full thickness ileal or colonic tissue strips (3 × 10 mm) were mounted along the longitudinal muscle orientation in individual muscle baths (Radnoti Glass, Monrovia, CA, USA) filled with 10 mL of carbogenated Krebs solution at 37 °C. The contractile activity was recorded as previously described [40,41], with isometric force transducers and amplifiers (Grass Instruments, West Warwick, RI, USA) connected to a Biopac data-acquisition system (Biopac Systems, Goleta, CA, USA). The muscle strips were equilibrated in the muscle bath under 1 g tension for 60 min at 37 °C before they were tested for contractility. Muscle contractility was tested in response to acetylcholine (ACh) (10^−7^ to 10^−3^ M), with each concentration being recorded for at least 2 min. The contractile response was quantified as the increase in area under contractions (AUC) during 2 min after the addition of each concentration of ACh over the baseline AUC for 2 min before the addition of the first concentration of ACh (10^−7^ M). 

To determine the direct effects of coffee and decaf on gut motor activity, ileal and colonic muscle strips (3 × 10 mm with the long axis along with the longitudinal muscle orientation) were isolated from naïve rats. The muscle contractile response to coffee or decaf (0.1~10 mg/mL) was determined with each concentration being recorded for at least 2 min. The contractile response was quantified as the increase in area under contractions (AUC) for 2 min after the addition of each concentration of coffee or decaf over the baseline AUC for 2 min before the addition of the first concentration (0.1 mg/mL). To study the mechanistic sites of action of coffee and decaf, the contractile response to coffee or decaf at 5 mg/mL was recorded in the absence and presence of the nicotinic receptor antagonist hexamethonium (Hex, 10^−4^ M), muscarinic receptor antagonist atropine (Atr, 10^−6^ M), and neurotoxin tetrodotoxin (TTX, 10^−6^ M) [42,43].

### 2.8. Statistical Analysis

Data points are expressed as means ± SEM, unless otherwise specified. A statistical analysis was performed by analysis of variance with non-repeated measures (by Student-Newman-Keuls test) for comparisons of multiple groups and Student’s t-test for comparisons of two groups. A *p* value of ≤0.05 was considered statistically significant. 

## 3. Results 

### 3.1. In Vitro effects of Regular and Decaffeinated Coffee on Gut Microbiota

**Figure 1:**
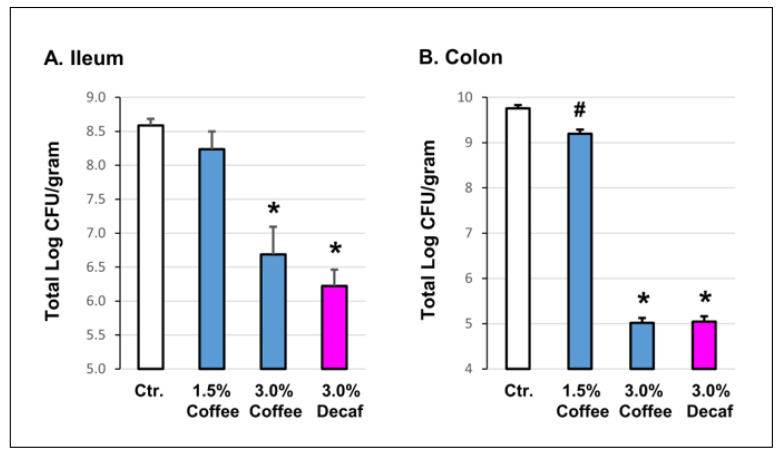
Inhibitory effect of coffee on gut microbiota growth in vitro. Serial dilutions of intraluminal fecal contents isolated from the ileum (5~10 cm from the ileum-colon junction, (**A**) and colon (5 cm from the end of colon, (**B**) of naïve rats were plated on regular LB agar and LB agar containing 1.5% coffee, 3.0% coffee, and 3.0% decaffeinated coffee (decaf). Bacterial colonies were counted 24 h later. N = 4 or 5 independent experiments, # *p* < 0.05 vs. control (Ctr.); * *p* < 0.001 vs. Ctr.

We first determined the in vitro effect of coffee on gut microbiota in a bacterial growth inhibition assay [18,26]. Intraluminal fecal contents were collected from the distal ileum in Figure 1A and colon in Figure 1B of naïve rats. The fecal contents were diluted and cultured on regular and coffee-containing LB agar plates. Compared to regular LB agar, the growth of bacteria from the intraluminal colon content was significantly (*p* = 0.009) suppressed in LB agar with 1.5% coffee (9.31 × 10^9^ CFU/gram vs. 9.65 × 10^9^ CFU/gram). With 3% coffee, the growth of the microbiome was further suppressed to 5.02 × 10^5^ CFU/gram, *p* = 0.000). A similar inhibitory effect was found for the ileal contents (Figure 1). 

To determine if the bacterial growth inhibition effect is caffeine-dependent or not, we cultured the colonic and ileal fecal contents in the agar containing 3% decaffeinated coffee. Interestingly, decaffeinated coffee had a similar inhibitory effect on the gut microbiota as regular coffee (Figure 1).

### 3.2. In Vivo Effects of Regular and Decaffeinated Coffee on Gut Microbiota

**Figure 2:**
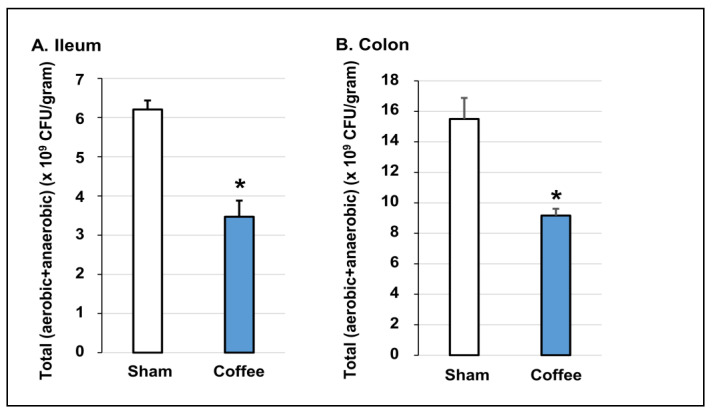
In vivo effect of coffee on gut microbiota in the ileum and colon determined by bacterial culture (anaerobic in GAM+ aerobic in TSA). Regular coffee solution (250 mg in 2 mL water) was administered by oral gavage daily for 3 days. Sham rats were treated similarly, but with 2 mL of saline. Rats were euthanized 3 days later (24 h after the 3rd coffee treatment). The intraluminal contents from the ileum (**A**) and colon (**B**) were collected. The ileal and colonic contents were cultured in anaerobic (GAM) and aerobic (TSA) conditions. The total bacteria abundance (CPU/gram of luminal contents) was counted. N = 4 or 5 rats. * *p* < 0.05 vs. sham.

To determine if regular daily consumption of coffee has any effect on the gut microbiota, we determined the in vivo effect of coffee gavage on the total bacteria population by culture and with qPCR techniques. We found that oral gavage treatment with coffee significantly suppressed the gut microbiome. After 3-days of treatment with coffee, the viable bacteria (counted in anaerobic and aerobic culture conditions separately) were decreased from 1.50 × 10^10^ to 9.16 × 10^9^ (CFU/gram of contents, *p* = 0.03) in the colon, and from 6.21 × 10^9^ to 3.47 × 10^9^ (CFU/gram of contents, *p* = 0.01) in the ileum (Figure 2). 

**Figure 3:**
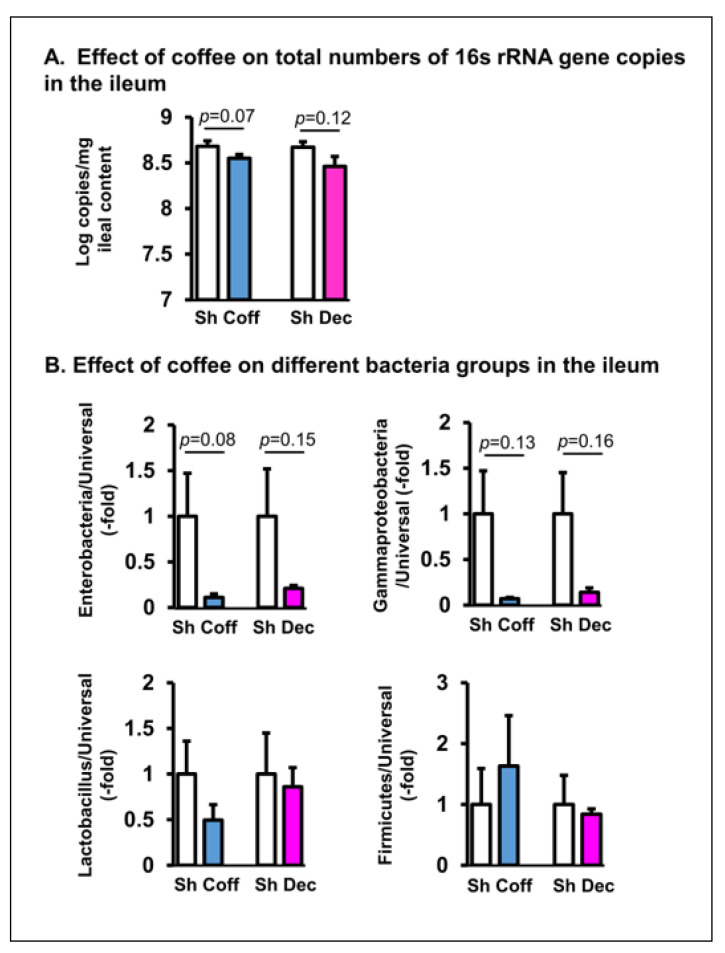
Quantitative RT-PCR detection of the in vivo effect of coffee on the total microbiota and different groups of microbes in the ileum. Coffee (Coff) or decaffeinated coffee (Dec) (250 mg in 2 mL water) was administered by oral gavage daily for 3 days. Sham rats were treated similarly, but with 2 mL of saline. Rats were euthanized 3 days later. The intraluminal contents from the ileum were collected. The total microbiota (**A**) and compositions of different groups (**B**) of microbes (Enterobacteria, Gammaproteobacteria, Lactobacillus, and Firmicutes) relative to overall microbiota (universal) were determined by real time PCR assays. N = 6 or 5 rats in each group. Sham (Sh). Coff, regular coffee; Dec, decaffeinated coffee.

**Figure 4:**
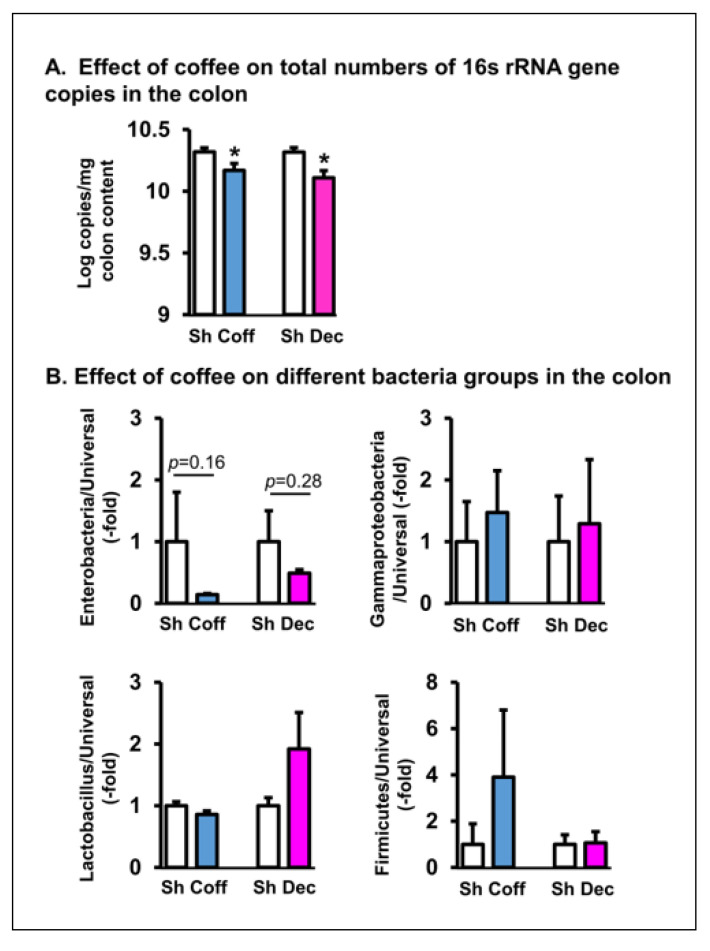
Quantitative RT-PCR detection of the in vivo effect of coffee on the total microbiota and different groups of microbes in the colon. Coffee (Coff) or decaffeinated coffee (Dec) (250 mg in 2 mL water) was administered by oral gavage daily for 3 days. Sham rats were treated similarly, but with 2 mL of saline. Rats were euthanized 3 days later. The intraluminal contents from the colon were collected. The total microbiota (**A**) and compositions of different groups of microbes (Enterobacteria, Gammaproteobacteria, Lactobacillus, and Firmicutes) relative to overall microbiota (universal) (**B**) were determined by real time PCR assays. N = 6 or 5 rats in each group. Sham (Sh). Coff, regular coffee; Dec, decaffeinated coffee. * *p* < 0.05 vs. sham of the group.

Consistent with the culture results, a qPCR study of gut microbiota in the ileum (Figure 3) and colon (Figure 4) found that coffee treatment significantly decreased the total bacteria abundance in the colon (*p* = 0.034. N = 7) and had the trend of suppressing bacteria counts in the ileum (*p* = 0.07. N = 7). The consumption of decaffeinated coffee had a similar inhibitory effect on the gut microbiota in vivo (Figure 3 and Figure 4). 

We further determined the effect of regular and decaffeinated coffee on different groups of gut microbes relative to the whole microbiota (Figure 3 and Figure 4). It appears that coffee mainly inhibits *Enterobacteriaceae* and *Gammaproteobacteria*, as it had a trend to further decrease the relative abundance of these groups in the ileum, but not of *Firmicutes* and *Lactobacillus* in both the small intestine and colon (*p* > 0.05. Figure 3 and Figure 4). Treatment with decaffeinated coffee had the similar effect as regular coffee on the different groups of bacteria in the colon and ileum. 

### 3.3. Effects of Consumption of Regular and Decaffeinated Coffee on Gut Motility and Smooth Muscle Contractility of the Small Intestine and Colon

**Figure 5:**
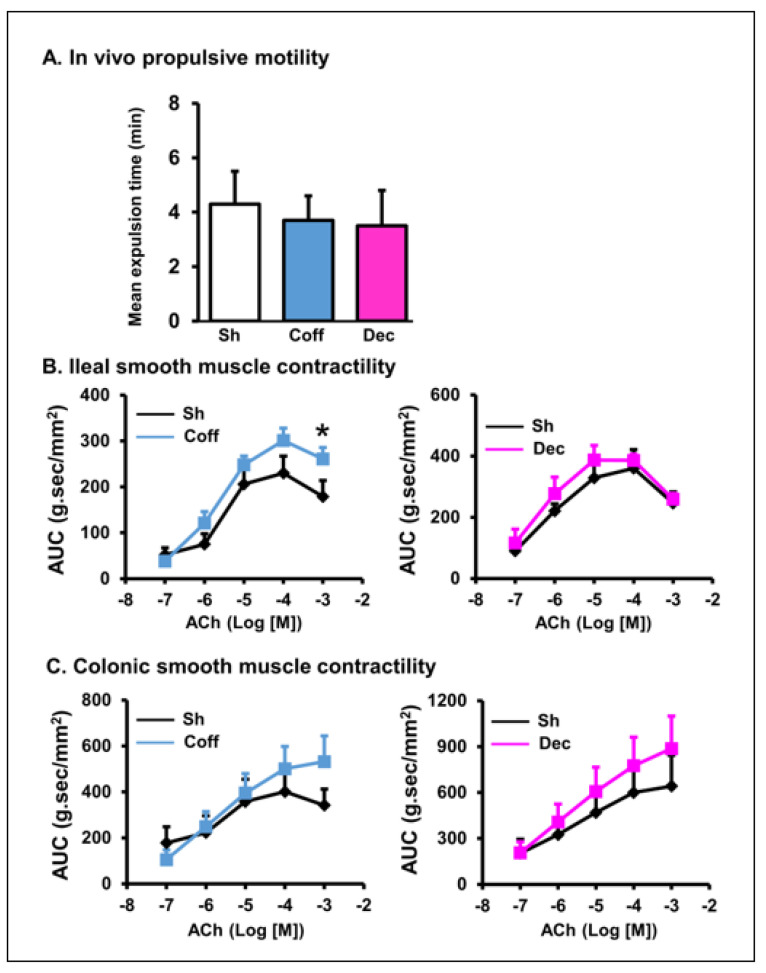
Effect of coffee on gut propulsive motility and smooth muscle contractility in the ileum and colon. Regular coffee (Coff, blue) and decaffeinated coffee (Dec, pink) (250 mg in 2 mL water) was administered by oral gavage daily for 3 days. Sham rats were treated similarly, but with 2 mL of saline. Rats were euthanized 3 days later. Colon motility was measured by counting the time needed to expel the inserted pellet from the anus (**A**). The ileal (**B**) and colonic (**C**) muscle strips were isolated from the rats, and their longitudinal muscle contractility was recorded in muscle bath. The smooth muscle contractile response to acetylcholine (ACh) was determined. N = 6 or 5 rats in each group. * *p* < 0.05 vs. sham. Sham (Sh). Coff, regular coffee; Dec, decaffeinated coffee.

To determine if coffee consumption has any long-term effect on gastrointestinal motility, we first measured the mean transit time of the colon to expel a pellet after rats were treated with coffee or decaf for 3 days (~24 h after the final gavage treatment). We found that the transit time was not statistically significant among the saline, coffee, and decaf groups (Figure 5A). We then compared the ileal and colonic smooth muscle contractility of rats treated with saline, coffee, and decaf. The ileal and colonic muscle strips were prepared along the longitudinal direction, and their contractile response was recorded in response to muscarinic cholinergic activation with acetylcholine (ACh, 10^−7^~10^−3^ M). Compared to vehicle controls, coffee treatment in vivo for 3 days did not affect muscle contractile response to ACh in the colon and ileum (Figure 5B,C), except that the response to one concentration (10^−3^ M) of ACh was statistically increased in the coffee treatment group compared to the vehicle group (Figure 5B, left panel). 

As with regular coffee, daily treatment with decaffeinated coffee did not show significant effects on the colonic and ileal smooth muscle contractile responses (Figure 5B,C). 

### 3.4. Direct Effect of Coffee on Gut Smooth Muscle Contractility

**Figure 6:**
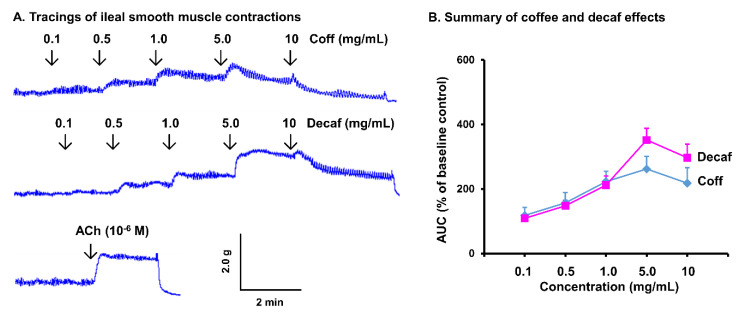
In vitro effect of coffee on ileal muscle contractions. Ileal muscle strips were isolated from naïve rats, and their longitudinal muscle contractility was recorded in muscle bath (**A**). The in vitro effects of regular coffee (Coff) and decaffeinated coffee (Decaf) at different concentrations (0.1~10 mg/mL) on muscle contractility in the first 2 min after each dose was measured (**B**). N = 4 independent experiments in 4 rats.

**Figure 7:**
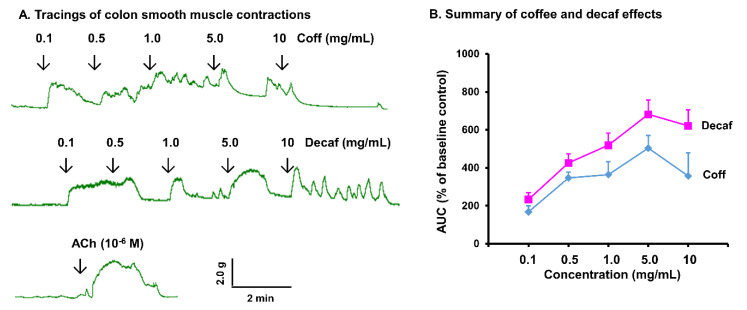
In vitro effect of coffee on colonic muscle contractions. Colonic muscle strips were isolated from naïve rats, and their longitudinal muscle contractility was recorded in muscle bath (**A**). The in vitro effects of regular coffee (Coff) and decaffeinated coffee (Decaf) at different concentrations (0.1~10 mg/mL) on muscle contractility in the first 2 min after each dose were measured (**B**). N = 4 independent experiments in 4 rats.

As coffee consumption did not show a significant genomic effect on gut smooth muscle contractility, we then detected the direct effect of coffee on ileal and colonic smooth muscle strips in a muscle bath study. Remarkably, coffee treatment in vitro induced a robust contractile response of the ileal and colonic smooth muscle in a dose-dependent manner (0.1 to 10 mg/mL) (Figure 6 and Figure 7). As an example, coffee at 1 mg/mL significantly increased the integral contractions of ileal and colon smooth muscle by 243 (±26)% and 478 (±45)% (*p* < 0.01 vs. control), respectively. Furthermore, decaffeinated coffee increased smooth muscle contractility to a similar extent as regular coffee. This demonstrates that the robust effect of coffee on intestinal and colonic smooth muscle contractions is in a caffein-independent manner. The contractile response to 1~5 mg/mL coffee or decaf is similar as to 10^−6^ M acetylcholine (Figure 6A and Figure 7A, bottom tracings), the prototype excitatory neurotransmitter in the neuromuscular transmission in the gut. 

### 3.5. Neuromuscular Mechanisms of Coffee Effect on Gut Smooth Muscle Contractions

**Figure 8:**
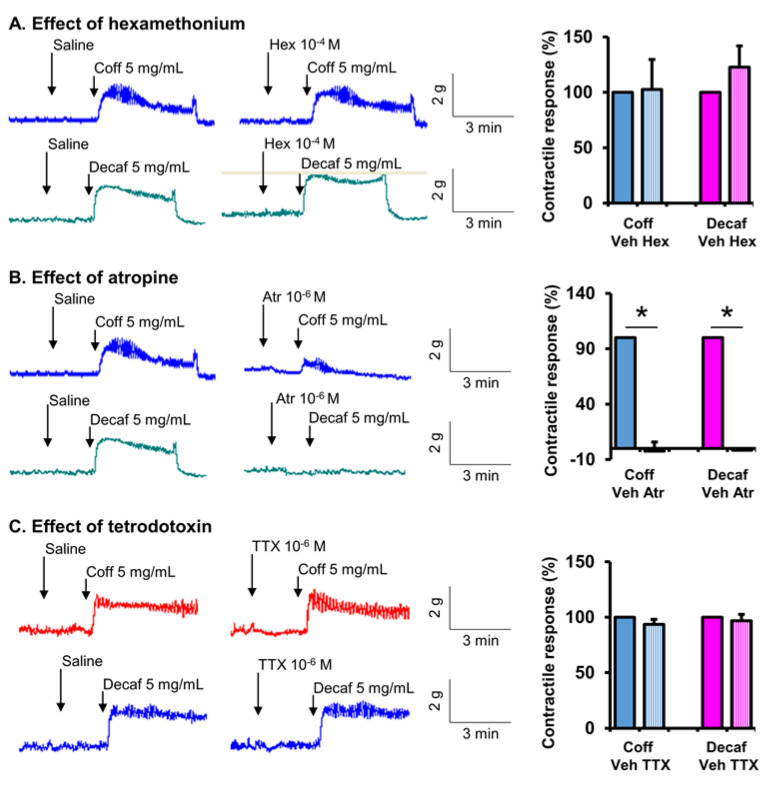
Site of action of coffee on smooth muscle contractions in the ileum. Ileal muscle strips were isolated from naïve rats, and their longitudinal muscle contractile activities were recorded in a muscle bath. The contractile response to regular coffee (Coff) and decaffeinated coffee (Decaf) at 5 mg/mL each was recorded in the absence and presence of nicotinic receptor antagonist hexamethonium (Hex, 10^−4^ M) (**A**), muscarinic receptor antagonist atropine (Atr, 10^−6^ M) (**B**), and neurotoxin tetrodotoxin (TTX, 10^−6^ M) (**C**). Tracings are representative of 4~5 independent experiments. The response in the first 2 min after the addition of coff or decaf over baseline activity is summarized in the bar graphs. N = 4 or 5. * *p* < 0.05.

**Figure 9:**
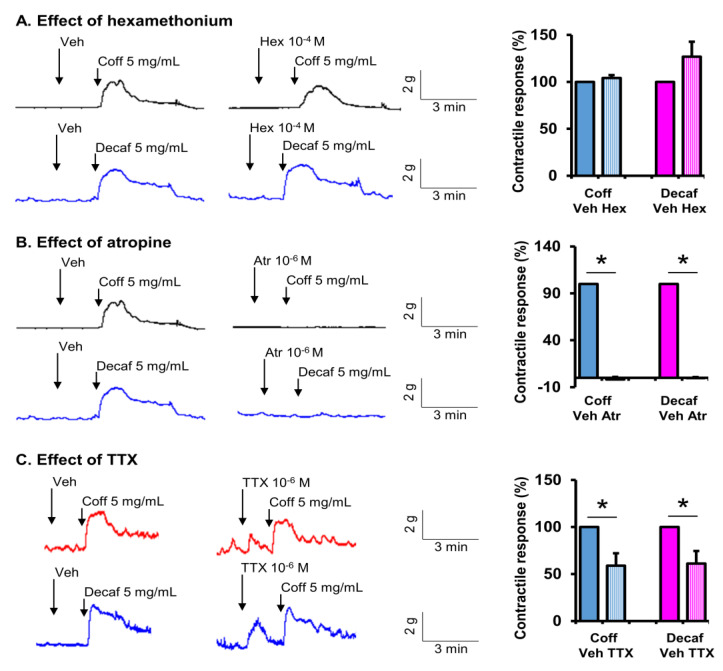
Determination of site(s) of action of coffee on smooth muscle contractions in the colon. Colonic muscle strips were isolated from naïve rats, and their longitudinal muscle contractile activities were recorded in the muscle bath. The contractile response to regular coffee (Coff) and decaffeinated coffee (Decaf) at 5 mg/mL was recorded in the absence and presence of the nicotinic receptor antagonist hexamethonium (Hex) (**A**), the muscarinic receptor antagonist atropine (Atr) (**B**), and the neurotoxin tetrodotoxin (TTX) (**C**). Tracings are representative of 4~5 independent experiments. The response in the first 2 min after the addition of coff or decaf over baseline activity is summarized in the bar graphs. N = 4 or 5. * *p* < 0.05.

We then investigated the neuromuscular mechanisms of action of coffee on smooth muscle contractions. Neural control of gut smooth muscle contractions is mainly through the excitatory motor neurons of intrinsic myenteric plexus of the enteric nervous system by releasing the neurotransmitter acetylcholine acting on cholinergic muscarinic receptors in smooth muscle cells [20,42]. The extrinsic nervous system (i.e., parasympathetic nervous system) indirectly controls smooth muscle contractions by innervating the myenteric ganglia through acetylcholine acting on nicotinic receptors in the enteric motor neurons. In our study, when cholinergic nicotinic receptor was blocked with hexamethonium (10^−4^ M), the contractile effect of coffee remained intact in the ileal and colon strips (Figure 8A and Figure 9A). However, when the cholinergic muscarinic receptor antagonist atropine (10^−6^ M) was present in the bath solution, the stimulating effect of coffee on muscle contractions was almost completely blocked (Figure 8B and Figure 9B). Hexamethonium and atropine had the same effect on decaffeinated coffee-induced contractions in the ileum and colon (Figure 8 and Figure 9). These results demonstrate that non-caffein component(s) in coffee solution acts on the intrinsic myenteric plexus or directly on smooth muscle cells to stimulate gut smooth muscle contractions in a muscarinic receptor-dependent mechanism. Interestingly, when neural activity is blocked with tetrodotoxin (TTX, 10^−6^ M), the coffee- or decaf-evoked contractile response in the ileal muscle remains intact (Figure 8C). However, TTX partially but significantly attenuated coffee- or decaf-evoked contractile response in the colonic muscle strips (Figure 9C). These data indicate that coffee or decaf contracts the ileal smooth muscle by acting directly on the cholinergic muscarinic receptor in the muscle cells. However, coffee or decaf acts on both myenteric neurons and the smooth muscle to contract the colonic smooth muscle in a muscarinic receptor-dependent mechanism.

## 4. Discussion

In previous attempts to understand the impact of coffee on the microbiota, Jaquet et al. [17] found that coffee might promote the growth of probiotic bacteria, while others [19] reported that coffee had no effect on fecal bacteria. Our study aimed to determine not only the in vivo effects of coffee on the gut microbiota in the small intestine and colon, but also the growth of microbiota in vitro. We first cultured the whole population of gut microbiota in the intraluminal (fecal) contents of the ileum and colon on regular LB agar and agar with coffee. Remarkably, we found that bacterial growth was suppressed by 100~1000-fold on coffee-agar, compared to regular LB agar. This antibacterial effect is not caffein dependent, as decaffeinated coffee had a similar inhibitory effect on the gut microbiota. To determine if coffee consumption has an anti-bacterial effect, we chose to deliver coffee brew to rats by oral gavage at 1 g/kg of body weight. This dose of coffee for a rat of ~250 g is similar to the amount of coffee consumed by a person with a body weight of 75 kg consuming 4 cups of coffee per day. Interestingly, we found that coffee treatment for three days substantially decreased total microbiota abundance in the ileum and colon, measured by either bacterial culture or quantitative RT-PCR. Again, this in vivo anti-bacterial effect appears to also be independent of caffein, as treatment with decaffeinated coffee achieved a similar inhibitory effect as regular coffee. To determine whether coffee has a similar inhibitory effect on harmful and beneficial gut bacteria, we chose to quantitate the abundance of four different groups of gut microbes, i.e., *Enterobacteriaceae, Gammaproteobacteria*, *Firmicutes* and *Lactobacillus*, in control and coffee treated rats. Interestingly, we found that regular coffee and decaffeinated coffee had a trend to further suppress the relative abundance of *Enterobacteriaceae* in the colon and ileum. However, neither regular coffee nor decaffeinated coffee had any inhibitory effect on *Firmicutes* or *Lactobacillus* in the small intestine and colon. *Enterobacteria* include some widely recognized pathogenic bacteria in the gut, i.e., *E. coli, Salmonella* and *Shigella*. A previous study by Nakayama and Oishi noted that coffee significantly inhibited *E. coli*, which belongs to *enterobacteria* [18]. On the other hand, *Firmicutes* and *Lactobacillus* are well known probiotics for their beneficial effects in the gut [14,27,44]. Taken together, our in vitro and in vivo results suggest that coffee has anti-bacterial properties in the gut. This anti-bacterial effect appears largely beneficial, as coffee is more inhibitory to potentially harmful bacteria such as *enterobacteria*, rather than to beneficial ones such as *Firmicutes* or *Lactobacillus*. 

It remains to be determined which components in coffee exerted the anti-bacterial action. However, our study found that decaffeinated coffee had a similar effect as regular coffee in inhibiting gut microbiota in vitro and in vivo. Thus, caffeine may not be the component exerting the anti-bacterial action. Among hundreds of bioactive components in coffee, several may have contributed to the anti-bacterial property. Chlorogenic acid (CGA), an important biologically active dietary polyphenol, is a major component of regular and decaffeinated coffee [45]. CGA is reported to not only have antioxidant and anti-inflammatory properties, but also antibacterial effects [46,47]. CGA has an inhibitory effect on both Gram-positive and Gram-negative bacteria by disrupting the cell membrane and interfering with the cell cycle and the metabolism of bacterial cells [46,48]. There are reports that coffee silverskin byproducts generated during the coffee roasting process may also have anti-microbial potential [49]. In addition, in vitro studies found that coffee melanoidins are also anti-bacterial, especially against *E. coli*, via a membrane damage mechanism [50]. 

Studies in healthy human subjects found that drinking coffee increased colon motility [22,23]. Surveys also showed that coffee drinking is associated with a decreased risk of constipation [5]. Furthermore, recent clinical trials found that drinking coffee during the post-operation period reduces the time to have first bowel movement and the incidence of post-operative ileus [7,9,10]. These studies suggest that coffee may have pro-motility properties. In the second part of the study, we aimed to determine if the pro-motility effect of coffee is via its genomic effect or immediate pharmacological action on the neuromuscular control of gut smooth muscle contraction. The administration of coffee for three days at a dose similar to that consumed daily by a human being did not significantly change gut smooth muscle contractility in the colon or ileum. This suggests that coffee may not have a significant genomic effect on the neuro-musculature of the GI tract (i.e., the up-regulation of genes encoding key biomolecules in neuromuscular transmission or contractile proteins involved in smooth muscle contractions). 

We then decided to investigate the pharmacological effects of coffee on gut smooth muscle contractility and its mechanism of action by testing the immediate action of coffee on the ileal and colon tissue strips. Our data showed that regular or decaffeinated coffee increased ileal and colonic smooth muscle contractility in a dose-dependent manner (0.1~10 mg/mL). The contractile response induced by 1 mg/mL coffee is similar to that induced by 1 µM of acetylcholine, a key neurotransmitter released from the myenteric motor neurons in the enteric nervous system to excite the gut smooth muscle for contractions. Our study thus indicates that the pro-motility effect of coffee is mainly through its immediate pharmacological effect on the neuro-musculature of the gut. 

We further determined the site of action of coffee on gut smooth muscle contractions. The neuro-muscular control of gut smooth muscle contractions involves pre-ganglia neural innervation via nicotinic receptors acting on the motor neurons in the myenteric ganglia. The excitatory motor neurons are primarily cholinergic, acting on cholinergic muscarinic receptors in gut smooth muscle cells leading to contractions [20,42]. In our study, pretreatment with the nicotinic receptor antagonist hexamethonium did not have any inhibitory effect on coffee-evoked contractile response, suggesting that coffee does not act on the site of pre-ganglia neural transmission. However, the cholinergic muscarinic receptor antagonist atropine almost completely blocked coffee-evoked contractions in the ileal and colonic muscle strips. This suggests that coffee exerts its contractile effect by acting on the post-ganglia neural pathway or directly on muscarinic receptors in gut smooth muscle cells. Moreover, when neural activity is blocked with TTX, the coffee-evoked contractile response in the ileal muscle remained intact. However, TTX significantly attenuated coffee-evoked the contractile response in the colonic muscle strips. These data indicate that coffee contracts ileal smooth muscle by acting directly on cholinergic muscarinic receptors in the muscle cells. However, it contracts colonic smooth muscle by acting on both myenteric neurons and smooth muscle in a muscarinic receptor-dependent mechanism. 

Interestingly, decaffeinated coffee acted exactly as coffee in its contractile action, suggesting that caffeine is not a key molecule in the coffee solution in exerting a contractile effect. In fact, caffeine was found to have an inhibitory effect on gut smooth muscle contractions at low doses [51]. At higher doses, caffeine may cause a transient contraction followed by the prolonged relaxation of the gut smooth muscle [52]. Our findings are supported by clinical observations that, like regular coffee, decaffeinated coffee caused increased colonic motor activity in normal subjects [22,23], and had a beneficial effect on post-operational ileus [8,10]. 

Currently, we do not know exactly which component(s) in the coffee solution is (are) responsible for the contractile response. Among the many bioactive components in coffee solution, melanoidins of the maillard reaction products, polyphenols, i.e., CGA and caffeic acid (CA), or choline may be among the key potential candidate molecules involved in the actions on gut smooth muscle contractions. Melanoidins such as Arg-Glu were found to lead to gastric muscle contractions [53]. CGA and CA were found to have protective effects on the enteric nervous system [24,54]. However, CA seems to lead to smooth muscle relaxation [55]. Choline, as a nutrient, is found in coffee, although in small amounts. The action of choline to cause gut smooth muscle contractions is well known [56]. Whether any of these potential candidate molecules is involved in the coffee effect on ileal and colonic smooth muscle contractions will be determined in our future study. Such studies are expected to better understand the health benefit of coffee and even to identify pharmacological targets for therapeutics for gut motility disorders. 

There are some limitations in our study. The results in the present article are based on our study with the specific regular and decaffeinated coffee at the doses and concentrations described in Materials and Methods. Although we tried another brand of roasted regular and decaffeinated coffee and found that their effects on microbiota and motility are similar to the results reported here, we are not certain if other types of coffee at different doses will achieve the same effects. In our in vivo study, we only studied the coffee effects for three days. We hope for the opportunity to study the effects of coffee on the gut over longer times in the future.

Taken together, our in vitro and in vivo studies show that coffee inhibits the gut microbiota in a caffeine-independent manner. The anti-bacterial effect of coffee is more effective for *Enterobacteria* than for beneficial bacteria such as *Firmicutes* and *Lactobacillus*. We found that coffee has a profound pro-motility effect on the ileum and colon, also in a caffein-independent manner. However, coffee exerts a pro-motility effect not through a genomic effect, as the administration of coffee for days did not change motility or muscle contractility. Rather, coffee has a robust pharmacologic effect to directly induce gut smooth muscle contractions in the ileum and colon. Mechanistic studies demonstrate that coffee acts on smooth muscle cells (in the small intestine) and post-ganglia neural sites (in the colon), rather than the pre-ganglia neural site, to evoke smooth muscle contractions in a muscarinic receptor-dependent mechanism.

## Data Availability

The raw data supporting the conclusion of this article are made available by the authors, without undue reservation.
